# Supplementary material for: Increase in clinically recorded type 2 diabetes after colectomy
Source: eLife. 2018 Oct 30;7:e37420. doi: 10.7554/eLife.37420 (PMC6207427; doi:10.7554/eLife.37420)
Supplement: Supplementary file 1. [file elife-37420-supp1.docx]

**Supplementary File 1.** Diagnoses for patients with colectomy who did not have colorectal cancer

| **Diagnoses** | **N (%) patients** |
| --- | --- |
| Diverticulosis | 5340 (32) |
| Inflammatory bowel disease | 2670 (16) |
| Polyps / Benign neoplasms | 1639 (10) |
| Paralytic or obstructive ileus | 1566 (9) |
| Other intestinal diseases | 1964 (12) |
| Other causes | 3477 (21) |
| Total | 16656 (100) |

Diagnosis grouping is based on the main diagnosis for the surgery hospital contact
