# Supplementary material for: Increase in clinically recorded type 2 diabetes after colectomy
Source: eLife. 2018 Oct 30;7:e37420. doi: 10.7554/eLife.37420 (PMC6207427; doi:10.7554/eLife.37420)
Supplement: Supplementary file 2. [file elife-37420-supp2.docx]

**Supplementary File 2.** Hazard ratio of clinically recorded type 2 diabetes after colectomy according to start of follow-up time

| **Start of follow-up time** | **Patients with colectomy**  **(T2D / total)** | **Non-colectomy patients**  **(T2D / total)** | **Hazard ratio**  **(95% CI)** | **P-value** |
| --- | --- | --- | --- | --- |
| **Total colectomy** |  |  |  |  |
| From date of surgery | 443 / 6,957 | 5,208 / 104,355 | 1.72 (1.56-1.89) | <.001 |
| 500 days after surgery | 246 / 4,575 | 2,873 / 68,625 | 1.44 (1.26-1.64) | <.001 |
| 1000 days after surgery | 201 / 3,793 | 2,323 / 56,895 | 1.40 (1.21-1.62) | <.001 |
| 1500 days after surgery | 167 / 3,256 | 1,886 / 48,840 | 1.42 (1.21-1.67) | <.001 |
| **Left hemicolectomy** |  |  |  |  |
| From date of surgery | 302 / 5,075 | 4,346 / 76,120 | 1.37 (1.22-1.54) | <.001 |
| 500 days after surgery | 176 / 3,218 | 2,390 / 48,260 | 1.33 (1.14-1.55) | <.001 |
| 1000 days after surgery | 145 / 2,513 | 1,754 / 37,685 | 1.41 (1.19-1.67) | <.001 |
| 1500 days after surgery | 120 / 1,986 | 1,278 / 29,780 | 1.55 (1.28-1.87) | <.001 |
| **Sigmoidectomy** |  |  |  |  |
| From date of surgery | 1,489 / 24,686 | 21,499 / 370,265 | 1.25 (1.18-1.31) | <.001 |
| 500 days after surgery | 977 / 17,159 | 13,214 / 257,370 | 1.26 (1.18-1.35) | <.001 |
| 1000 days after surgery | 829 / 13,927 | 10,504 / 208,880 | 1.30 (1.21-1.40) | <.001 |
| 1500 days after surgery | 668 / 11,486 | 8,194 / 172,290 | 1.31 (1.21-1.42) | <.001 |

Total colectomy includes colectomy and proctocolectomy. Hazard ratios are adjusted for age, sex, and year of surgery. T2D: clinically recorded type 2 diabetes.
