# Supplementary material for: Increase in clinically recorded type 2 diabetes after colectomy
Source: eLife. 2018 Oct 30;7:e37420. doi: 10.7554/eLife.37420 (PMC6207427; doi:10.7554/eLife.37420)
Supplement: Supplementary file 3. [file elife-37420-supp3.docx]

**Supplementary File 3.** Procedure codes for colectomies and proctectomies used to identify patients in the Danish National Patient Register

|  | **NCSP codes** | **DOTC codes** |
| --- | --- | --- |
| Total colectomy | KJFH00-KJFH11, KJFH20-KJFH40 | 45080, 45060-45065 |
| Right hemicolectomy | KJFB30-KJFB31 | 44900-44901 |
| Resection of colon transversum | KJFB40-KJFB41 | 44940 |
| Left hemicolectomy | KJFB46-KJFB47 | 44920-44921, 44960-44961 |
| Sigmoidectomy | KJFB46-KJFB47, KJFB60-KJFB61 | 44980-44981 |
| Proctectomy | KJGB00-KJGB11, KJGB30-KJGB50,  KJGB96-KJGB97 | 45540, 45700-45720, 45800-45840, 45845-45881, 46061 |

NSCPS: the Nordic Classification of Surgical Procedures was used from 1996 and onwards: <https://norden.diva-portal.org/smash/get/diva2:970547/FULLTEXT01.pdf>

DOTC: the Danish Surgical Procedure and Treatment Classification version 3 was used from 1994-1995: <ftp://filer.sst.dk/filer/sks/data/skscomplete/OPRklass_1995.txt>
