# Supplementary material for: Increase in clinically recorded type 2 diabetes after colectomy
Source: eLife. 2018 Oct 30;7:e37420. doi: 10.7554/eLife.37420 (PMC6207427; doi:10.7554/eLife.37420)
Supplement: Supplementary file 4. [file elife-37420-supp4.docx]

**Supplementary File 4.** Procedure codes used to identify non-colectomy patients in the Danish National Patient Register

|  | **NCSP codes** | **DOTC codes** |
| --- | --- | --- |
| Orthopedic surgery | N* | 70000-85899 |
| Abdominal surgery leaving the gastrointestinal tract intact | JA, JB, JC, JH | 40020-40200, 40220-40990  40000-41691, 46320-46990 |
| Other surgery, unrelated to the gastrointestinal tract | BA, BB, BC, G*, JM, K*, L* | 08000-08590, 08600-08890, 34020-35890, 48900-48950, 50020-58890, 60020-60890,  60920-61890, 61920-62590,  62620-62990, 63320-63390,  63420-63790, 63800-63890 |

NSCPS: the Nordic Classification of Surgical Procedures was used from 1996 and onwards: <https://norden.diva-portal.org/smash/get/diva2:970547/FULLTEXT01.pdf>

DOTC: the Danish Surgical Procedure and Treatment Classification version 3 was used from 1994-1995: <ftp://filer.sst.dk/filer/sks/data/skscomplete/OPRklass_1995.txt>
