# Supplementary figures and images for: Increase in clinically recorded type 2 diabetes after colectomy
Source: eLife. 2018 Oct 30;7:e37420. doi: 10.7554/eLife.37420 (PMC6207427; doi:10.7554/eLife.37420)

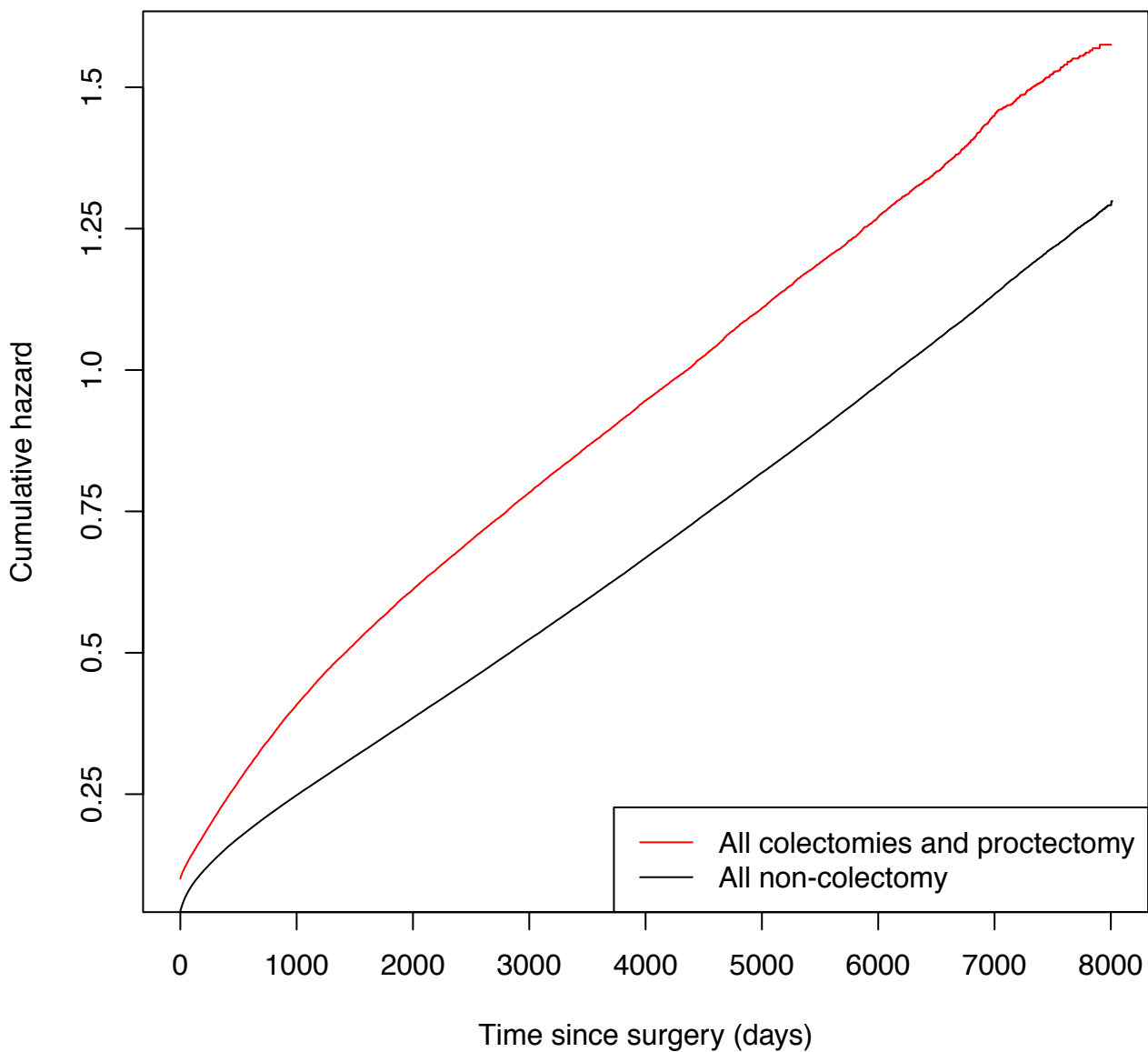

Supplement: Supplementary file 5. [file elife-37420-supp5.pdf]
